# Uncommon Association Between Gastrointestinal Stromal Tumors (GIST) and Pheochromocytoma With Abdominal Wall Relapse: Case Report and Literature Review

**DOI:** 10.7759/cureus.54532

**Published:** 2024-02-20

**Authors:** Cesar A Nieves Perez, Miguel C Molina Obana, Regina Uribe Torres, Sandra Rivera Delgado, Benito Ceballos Vazquez Tagle

**Affiliations:** 1 Internal Medicine, Hospital Angeles Pedregal, Mexico City, MEX; 2 General Physician, Universidad Anáhuac México, Mexico City, MEX

**Keywords:** gist, relapse, surgery, general internal medicine, gastrointestinal tumor (gist), gastrointestinal stromal tumor (gist)

## Abstract

Gastrointestinal stromal tumors (GISTs) represent a rare form of gastrointestinal neoplasm. This report details a medical case involving a 44-year-old woman who underwent bilateral pheochromocytoma resection, GIST gastrectomy, and laparoscopic adrenalectomy with intestinal resection. Despite an initially positive response to oral imatinib, treatment was delayed due to economic constraints. This delay resulted in a critical event marked by abdominal GIST metastasis to the abdominal wall, subsequent rupture leading to hemoperitoneum, and emergency surgery. Following an adequate postsurgical recovery, she was successfully discharged prior to medication adjustments.

## Introduction

Gastrointestinal stromal tumors (GISTs) are rare mesenchymal neoplasms, accounting for 1-2% of gastrointestinal tumors, arising from the Cajal intestinal cells responsible for gastrointestinal tract motility [1]. They usually present as subepithelial neoplasms in the stomach (56%), small intestine (32%), colon and rectum (6%), esophagus (0.7%), and, rarely, in the omentum, mesentery, peritoneum, or abdominal wall [2]. The GISTs can result from germline mutations and include various molecular entities with mutations in oncogenes, mainly KIT and PDGFRA [3]. Most GISTs are sporadic with no known risk factors; however, conditions like germline mutations in KIT, PDGFRA, or SDH genes, or loss of NF1 germline, are associated with higher risk [1,2].

The GISTs are often incidental findings during abdominal imaging or surgical procedures for other reasons. They are diagnosed through a combination of imaging modalities, biopsies with immunohistochemical testing, and genetic sequencing [4]. The introduction of tyrosine kinase inhibitors (TKIs) has significantly improved the morbidity and mortality of GISTs [3,4].

## Case presentation

This involves a 44-year-old woman with a history of bilateral resection of two pheochromocytomas in 2017, gastrectomy for a GIST tumor in the gastric antrum in 2019, with positive immunohistochemistry results for DOG1, CD117, CD34, Ki67 (5%), negative for S100 proteins, and positive for H-Caldesmon. Additionally, she underwent laparoscopic adrenalectomy plus intestinal resection and anastomosis of a 30 cm ileum for internal hernia in 2020.

**Figure 1:**
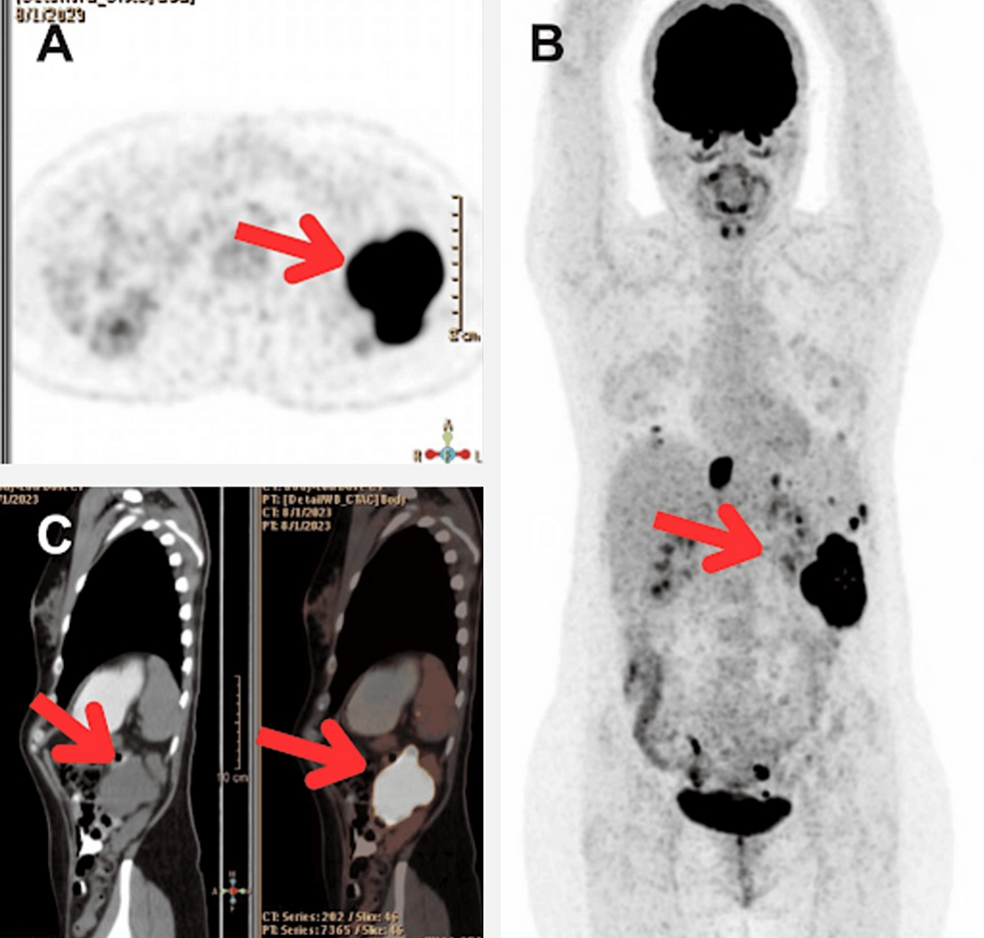
Abdominal positron emission tomography-computed tomography (PET-CT) In the three panels (A: axial in grayscale, B: coronal in grayscale, and C: simple phase contrast in hot metal scale), a left flank topography reveals the presence of a space-occupying lesion with lobulated contours, partially defined, in close contact with loops of the small intestine (proximal ileum and distal jejunum) and the spleen. The lesion measures 106 x 63 x 100 mm, with a volume of 350 cm³, and exhibits heterogeneous density, showing areas with hematic density (average attenuation of 75 HU), with suggestive images of peritoneal implants.

**Figure 2:**
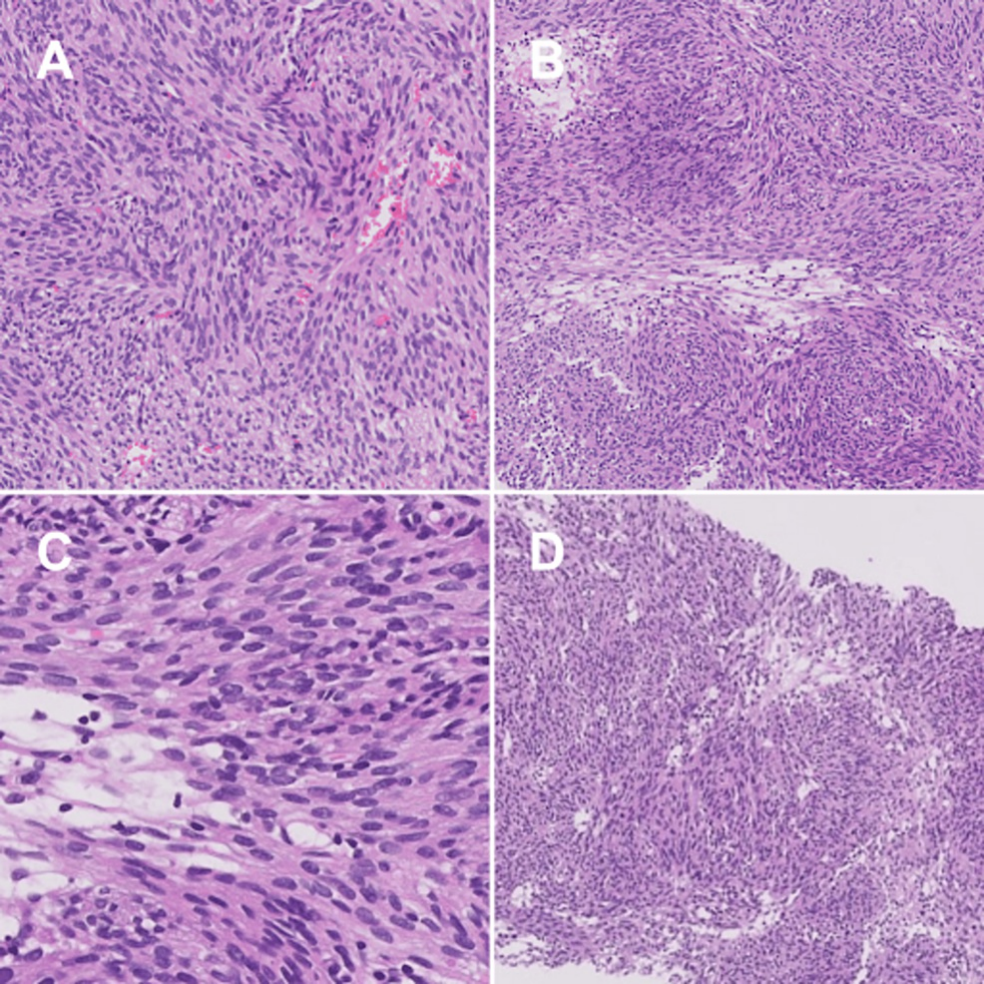
Radio-interventional tumor biopsy The histological sections with hematoxylin and eosin reveal a spindle-cell and epithelioid mesenchymal lesion, consisting of cells with elongated nuclei, eosinophilic cytoplasm, and small vacuoles. There is a mitotic index exceeding 5 in 5mm², and a focal area of necrosis measuring less than 1mm². Positive immunohistochemistry results for DOG1, CD117, CD34, Ki67 (5%), negative for S100 proteins, and positive for H-Caldesmon.

She presented to the emergency department with progressive and diffuse abdominal pain (Visual Analog Scale 6/10), radiating to the subscapular region, worsening with movements, and alleviated by an antalgic position, accompanied by episodes of vomiting. The vital signs revealed a blood pressure of 110/63 mmHg, heart rate of 89 bpm, oxygen saturation of 95%, temperature of 36.5°C, and respiratory rate of 22 rpm. Due to her history, a positron emission tomography with FDG (Figure 1) was performed, revealing metastatic lesions in the liver, spleen, and left paracolic gutter. An abdominal radio-interventional biopsy confirmed a mixed fusocelular and epithelioid gastrointestinal stromal tumor (GIST) with high mitotic activity (Index >5 mitoses in 5mm², with focal necrosis) (Figure 2). Immunohistochemistry confirmed the metastatic nature of GIST with CD117+, DOG1+, and CD34+, and a Ki67 proliferation index of 25%. The patient was discharged with a prescription for oral imatinib 400 mg every 24 hours. However, due to economic limitations, treatment initiation was delayed for two months.

**Figure 3:**
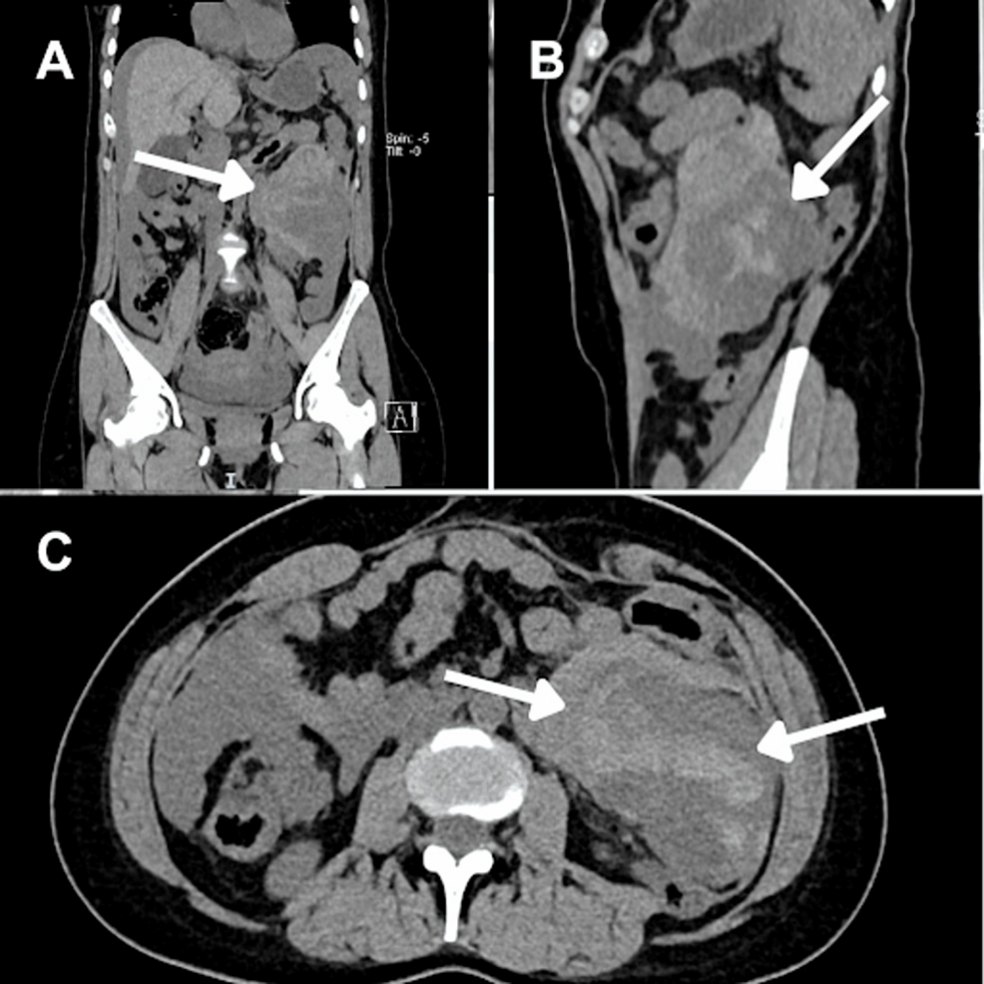
Abdominal computer tomography In the three panels (A: coronal, B: sagittal, and C: axial), a left flank topography reveals a space-occupying lesion with lobulated contours in close proximity to the small intestine. It measures 9.5x5.6x8.4 cm with a volume of 234 cm³. The lesion exhibits areas of hepatic density in the simple phase within the tumor.

**Figure 4:**
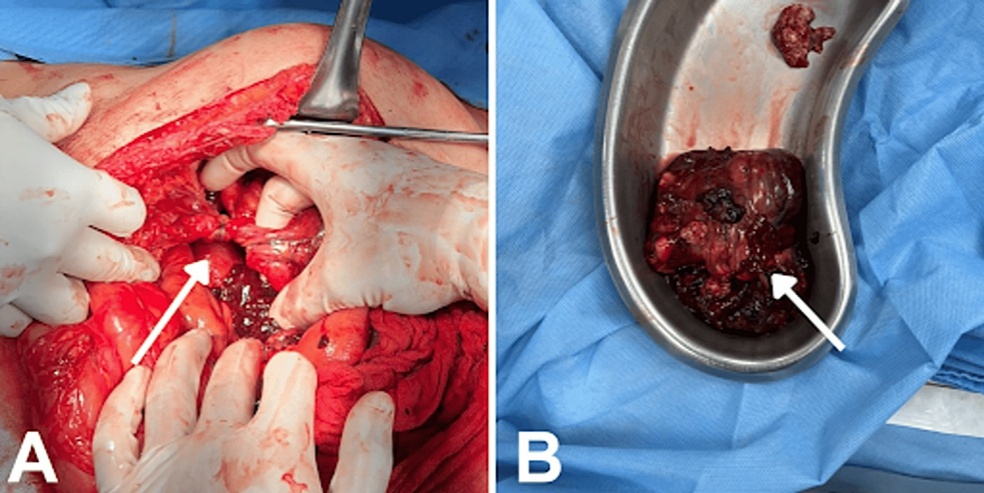
Macroscopic images of the tumor during surgery and post-resection In Panel A, the GIST tumor is observed attached through a twisted peduncle to the abdominal wall, greater omentum, and small intestine. In Panel B, the presence of the resected tumor is observed, with a broken peduncle at its base, as well as an area of intratumoral necrosis. The dimensions of the tumor measure 25x20x15 cm.

Two weeks after starting imatinib, the patient experienced severe abdominal pain (Visual Analog Scale 8/10) and sought emergency care. In the emergency department, vital signs revealed a blood pressure of 90/58 mmHg, heart rate of 77 bpm, oxygen saturation of 95%, temperature of 36°C, and respiratory rate of 20 rpm. Resuscitation with crystalloids was initiated, and an abdominal computed tomography (Figure 3) showed an increase in the infiltrative lesion of the proximal ileum with intralesional bleeding, subhepatic, perihepatic, and perisplenic free fluid, as well as infiltration towards the spleen and left subphrenic implants. The patient was admitted to an intermediate care unit with a hemoglobin of 10.4 on admission and 7.9 the next day. Immediate exploratory laparotomy was decided, revealing the rupture of the abdominal GIST metastasis, hemoperitoneum, and adhesions (Figure 4). Two liters of hemoperitoneum were found, along with a tumor adhered to the greater omentum and the abdominal wall, measuring 25x20x15 cm, ruptured and twisted at its base pedicle. The tumor was removed from its pedicle and there was no need for lymph node resection. The patient recovered without complications after surgery and was discharged with oral imatinib 400 mg every 24 hours, continuing the baseline treatment that she could not initially receive. In the following eight months of follow-up, there have been no adverse effects of the medication or recurrence of the GIST.

## Discussion

Epidemiology

Gastrointestinal stromal tumors (GISTs) account for approximately 1-2% of gastrointestinal neoplasms, making them the most common mesenchymal tumors of the gastrointestinal tract [1]. Globally, the incidence of GISTs ranges from six to 22 cases per million individuals per year, with variations based on region and time [4]. The majority of GISTs are diagnosed around the age of 60, with an equal distribution between genders [5]. Most cases present symptoms, and the most common anatomical locations are the stomach (55%), small intestine (31.8%), colorectal (6%), and others (5.5%). The highest prevalence is observed in China, Taiwan, and Norway, with an incidence of 19-22 cases per million inhabitants [6]. The majority of GISTs are unifocal (92%), with a histological composition of 75% fusiform cell type, 17.1% mixed epithelial and fusiform, and 7.8% epithelioid type [5,7].

Etiology

Gastrointestinal stromal tumors (GISTs) predominantly derive from the lineage of interstitial cells of Cajal, which serve as gastrointestinal pacemakers and are responsible for their motility. Although GISTs can originate from other cell types due to specific mutations, such as telocytes with mutations in PDGFRA or smooth muscle cells with mutations in BRAF, different exclusive and coexisting mutations may guide the development and presentation, both clinically and anatomically, of tumors [1,8].

The most common mutations observed are in KIT (60-70%) and PDGFRA (10-15%). These two genes encode a receptor for tyrosine kinase III. The KIT protein, encoded by the C/kit gene, is located on chromosome 4q11-12, and the PDGFRA protein is encoded by the PDGFRA gene on chromosome 4q11-12; both are proteins of the tyrosine kinase receptor family [1,8]. When mutated, they express abnormal activation that initiates intracellular pathways through phosphorylation. They express elevated levels of ETV1, a transcription factor necessary for the development of interstitial cells of Cajal (ICC) and for the proliferation of GIST cells. ETV1 is regulated by the activation of the MEK-MAPK pathway, which is under the regulation of the activated KIT and PDGFRA. The joint expression of activated KIT and ETV1 promotes the oncogenic transcription program necessary to transform ICC into GIST cells, with unregulated growth, proliferation, and inhibition of apoptosis [8,9].

Histologically, GISTs vary primarily by location. They usually present as well-defined tumors in the subserosa or submucosa of the gastrointestinal tract, exhibiting cytomorphological characteristics such as fusiform (70%), epithelioid (20%), or mixed (10%) features [10]. Gastric GISTs typically have a solid form with hyalinized stroma, indicating a myxoid component. They also exhibit perinuclear vacuolization, a finding that reinforces their relationship with Cajal cells. In the small intestine, they often have a more fusiform presentation, with a paragangliomatous pattern observed. They contain eosinophilic structures composed of collagen, which stain brightly with periodic acid-Schiff (PAS) staining [5].

Extra-gastrointestinal stromal tumors (E-GISTs), as in this case in the abdominal wall, generally share morphological, immunohistochemical, and molecular characteristics similar to conventional GISTs. It is probable that their origin is from ectopic interstitial cells of Cajal, derived from pluripotent mesenchymal cells [10]. Hajar et al. analyzed, through a case report, abdominal recurrences of a GIST following the resection of the primary tumor through laparoscopic procedures. They proposed mechanisms such as elevated CO2 insufflation, secondary dissemination due to inadequate protection of the abdominal wall during extraction, and excessive manipulation of the tumor during surgery, combined with their fragile nature due to necrosis [11].

The coexistence of pheochromocytoma and gastrointestinal stromal tumors (GIST) is rare; this association may be due to a common genetic pathway. However, the specific relationship between pheochromocytoma and GIST remains unclear [3]. In some cases, this relationship can be due to some genetic syndromes like Carney-Stratakis syndrome, also known as dyad or triad syndrome, which is a rare genetic condition characterized by the presence of both paragangliomas (including pheochromocytomas) and gastrointestinal stromal tumors (GISTs). It is an autosomal dominant disorder caused by mutations in genes associated with the succinate dehydrogenase (SDH) complex, particularly SDHB, SDHC, and SDHD genes. Recognizing and diagnosing Carney-Stratakis syndrome is important for appropriate management, including surveillance for tumor recurrence and genetic counseling for affected individuals and their families [1].

Clinical presentation

These tumors are often found incidentally during abdominal CT scans or surgical procedures for other reasons. In 18% of cases, GISTs may be asymptomatic, especially in cases of small tumors in the intestinal tract [4,5]. Symptomatic patients may experience nonspecific symptoms such as nausea, vomiting, abdominal distension, early satiety, abdominal pain, and rarely palpable abdominal mass. Large tumors can cause gastrointestinal lumen obstruction, leading to dysphagia, obstructive jaundice, or constipation, depending on the location of the mass. Perforated neoplasms present signs of peritonitis or gastrointestinal bleeding; massive or indolent intraperitoneal bleeding is secondary to pressure necrosis and ulceration [2,4].

Diagnosis

The typical presentation of GISTs is that of a gastrointestinal-origin tumor, with general symptoms in this organ. The approach to this pathology will necessitate integrating various diagnostic tests to confirm the diagnosis. In some cases, it may be found incidentally during ultrasound, CT scans, or endoscopies [1,4,12].

As the primary imaging diagnostic modality initially, we have CT, which is useful for detecting the tumor, planning surgical events, post-surgical surveillance, or even monitoring tyrosine kinase inhibitor management. Magnetic resonance imaging (MRI) is another important method, and although it typically obtains morphological imaging results similar to CT, its ability to obtain enhancement degrees, diffusion coefficients, and perfusion can provide more benefit in evaluating metastases and their response to treatment. Particularly, one of its advantages is the ability to detect liver metastases that may not be detected by CT [4,12].

Conventional ultrasound is used for two specific situations; first, the detection of masses or liver metastases, and secondly, it is used in interventional radiology methods for biopsies. One of the most useful studies is positron emission tomography with fluorodeoxyglucose (FDG-PET), which allows the visualization of metabolically active tissue, distinguishing between GIST and non-GIST tumors. Moreover, it provides the ability to evaluate tyrosine kinase inhibitor therapy and can predict the treatment response better than other imaging methods. One disadvantage of this method is that it may cause false positives by demonstrating increased uptake of inflammatory tissue in the context of the early postoperative evaluation period. Endoscopy, although limited in its role in pathology, has the advantage of having both a therapeutic and diagnostic role. It can take biopsies, and in the case of gastrointestinal bleeding associated with the neoplasm, it can control bleeding through hemostasis [1,4,5,12].

Tissue analysis is necessary for confirming the diagnosis. During microscopic morphological examination, three main histopathological patterns are shown: 70% of GISTs are of the fusiform cell type, characterized by cells with pale eosinophilic fibrillar cytoplasm, uniform ovoid nuclei, and poorly defined cell borders; 20% of GISTs are of the epithelioid type, composed of rounded cells with eosinophilic to clear cytoplasm arranged in sheets and nests. Finally, 10% are of the mixed type, showing both fusiform and epithelial cells. The morphological diagnosis can be supported using immunohistochemical methods. To continue with the definitive diagnostic protocol and achieve guided therapy, immunophenotype methods and mutation analysis will be used [1,4].

Treatment

Surgical treatment remained a key intervention for the management of early GIST, with a success rate of 60% in the pre-imatinib era [13]. The introduction of tyrosine kinase inhibitors revolutionized the treatment of GIST tumors due to their impressive and effective control of the disease. Imatinib is the current first-line treatment, especially in cases of KIT-and PDGFRA-sensitive GIST. Sunitinib and regorafenib are considered second or third-line treatments for patients with GIST who develop sensitivity to imatinib [14].

For the treatment of pheochromocytomas, the Endocrine Society recommends minimally invasive adrenalectomy, such as laparoscopy, for the treatment of most pheochromocytomas. Open resection should be reserved for large (>6 cm) or invasive pheochromocytomas to ensure complete tumor resection and prevent tumor rupture [15].

## Conclusions

Gastrointestinal stromal tumors (GISTs) are a rare type of gastrointestinal neoplasm, and their risk and etiological factors are not fully understood. This neoplasm may be related to various genetic syndromes with a wide variety of clinical presentations. Over the last decades, the treatment of these tumors has advanced by complementing surgical approaches with the introduction of tyrosine kinase inhibitors. In certain rare circumstances, as seen in our case, recurrence not only occurs but also originates from an abnormal site of presentation. This necessitates a new approach to the patient and the exclusion of other causes of the disease. Some other case reports attribute the origin of these abdominal implants to the surgical procedure, prompting the exploration of preventive measures during manipulation. 

The particular case of our patient's clinical presentation suggests, by its characteristics, a probable genetic syndrome in conjunction with inadequate administration of the established medication for the disease and a laparoscopic resection resulting in a relapse due to metastasis in the abdominal wall. We must comprehensively manage our patients and be aware of the multiple factors that can precariously affect our patients' development, thus taking preventive and even diagnostic measures of greater scope if necessary.
